# Phylogenetic analysis of mitochondrial protein coding genes confirms the reciprocal paraphyly of Hexapoda and Crustacea

**DOI:** 10.1186/1471-2148-7-S2-S8

**Published:** 2007-08-16

**Authors:** Antonio Carapelli, Pietro Liò, Francesco Nardi, Elizabeth van der Wath, Francesco Frati

**Affiliations:** 1 Department of Evolutionary Biology, University of Siena, via A.Moro 2, 53100, Siena, Italy; 2 The Computer Laboratory, University of Cambridge, William Gates Building, 15 JJ Thomson Avenue, Cambridge CB3 0FD, UK

## Abstract

**Background:**

The phylogeny of Arthropoda is still a matter of harsh debate among systematists, and significant disagreement exists between morphological and molecular studies. In particular, while the taxon joining hexapods and crustaceans (the Pancrustacea) is now widely accepted among zoologists, the relationships among its basal lineages, and particularly the supposed reciprocal paraphyly of Crustacea and Hexapoda, continues to represent a challenge. Several genes, as well as different molecular markers, have been used to tackle this problem in molecular phylogenetic studies, with the mitochondrial DNA being one of the molecules of choice. In this study, we have assembled the largest data set available so far for Pancrustacea, consisting of 100 complete (or almost complete) sequences of mitochondrial genomes. After removal of unalignable sequence regions and highly rearranged genomes, we used nucleotide and inferred amino acid sequences of the 13 protein coding genes to reconstruct the phylogenetic relationships among major lineages of Pancrustacea. The analysis was performed with Bayesian inference, and for the amino acid sequences a new, Pancrustacea-specific, matrix of amino acid replacement was developed and used in this study.

**Results:**

Two largely congruent trees were obtained from the analysis of nucleotide and amino acid datasets. In particular, the best tree obtained based on the new matrix of amino acid replacement (MtPan) was preferred over those obtained using previously available matrices (MtArt and MtRev) because of its higher likelihood score. The most remarkable result is the reciprocal paraphyly of Hexapoda and Crustacea, with some lineages of crustaceans (namely the Malacostraca, Cephalocarida and, possibly, the Branchiopoda) being more closely related to the Insecta *s.s*. (Ectognatha) than two orders of basal hexapods, Collembola and Diplura. Our results confirm that the mitochondrial genome, unlike analyses based on morphological data or nuclear genes, consistently supports the non monophyly of Hexapoda.

**Conclusion:**

The finding of the reciprocal paraphyly of Hexapoda and Crustacea suggests an evolutionary scenario in which the acquisition of the hexapod condition may have occurred several times independently in lineages descending from different crustacean-like ancestors, possibly as a consequence of the process of terrestrialization. If this hypothesis was confirmed, we should therefore re-think our interpretation of the evolution of the Arthropoda, where terrestrialization may have led to the acquisition of similar anatomical features by convergence. At the same time, the disagreement between reconstructions based on morphological, nuclear and mitochondrial data sets seems to remain, despite the use of larger data sets and more powerful analytical methods.

## Background

With over one million recognized species, which account for more than 80% of described animal species, Arthropoda (insects, crustaceans and their kin) display an unprecedented richness and extraordinary diversity in terms of morphology and lifestyle adaptations. Early differentiation of stem lineages, explosive radiations, and abrupt modifications in developmental patterns have been proposed to be responsible for such a diversity [1,2]. This, together with the subsequent long anagenetic evolution along each major lineage, has considerably complicated our possibility to reconstruct their phylogenetic relationships. Despite the fact that the interpretation of the evolutionary history of arthropod groups have long attracted the interest of systematists, relationships among and between major lineages are still fiercely debated.

Important contributions recently revolutionized the current view on the phylogenetic relationships among and within major lineages of Arthropoda (Chelicerata, Crustacea, Hexapoda and Myriapoda). One of the most contentious issues was the identification of the closest relative to the Hexapoda, with Myriapoda and Crustacea alternatively emerging as plausible candidates. While the traditional view, mostly based on morphological evidence, emphasized the affinities between Hexapoda and Myriapoda (= Atelocerata), recent molecular data consistently indicate crustaceans (or some of their lineages) as the sister group of the hexapods (Pancrustacea, *sensu *[3], or Tetraconata, *sensu *[4]) [5-11], with myriapods emerging earlier from the arthropod tree, or else associated with the chelicerates (Paradoxopoda, *sensu *[12], or Myriochelata *sensu *[10]). This has fostered a reappraisal of the morphological evidence [13,14], and the "Pancrustacea" hypothesis has gained growing credibility among the community of arthropod systematists.

Focusing on the relationships inside the Pancrustacea, recent phylogenetic reconstructions, based on the analysis of mitochondrial and nuclear genes, have questioned the mutual monophyly of crustaceans and hexapods [15,16]. These analyses led to a radical rearrangement of major pancrustacean lineages, with insects frequently emerging as a nested clade within crustaceans [12,17], and Branchiopoda and Malacostraca as the sister groups to hexapods, with the exclusion of other crustaceans [16,18]. In addition, the position of some basal groups, generally considered to be the earliest offshoots of hexapod evolution, has been questioned on the basis of molecular evidence, that suggests that some crustacean groups might be more closely related to the crown group of hexapods (Insecta *s.s*.) than Collembola and Diplura [19,20].

Hexapoda have been traditionally considered a monophyletic taxon based on the shared organization of body segments, the presence of six legs, and terrestrialization. According to mouthpart organization, two groups are generally recognized: the Entognatha (Protura, Collembola and Diplura) and the Ectognatha (Microcoryphia, Zygentoma and Pterygota) [21,22]. While the monophyly of Ectognatha (= Insecta *sensu stricto*, hereafter Insecta) seems to be reasonably well established, a coherent agreement for the relative position of Protura, Collembola and Diplura, and for the monophyly of this latter taxon, is not yet emerging [23-27]. Morphological or combined analyses [26,28], as well as analyses based on rRNA sequences [17,29,30], support a sister group relationship between Protura and Diplura (Nonoculata) with the exclusion of Collembola. Alternatively, molecular analyses based on mitochondrial genes consistently recover Collembola, and possibly Diplura, as emerging very early in the pancrustacean tree, with some crustaceans being more closely related to the Insecta than are the entognathans [15,16,19,31,32]. Mitochondrial gene order rearrangements, which proved themselves crucial for high-level phylogenetics (see [6] for a key example), do not seem to be informative at this level [20], although they might provide useful information within orders.

The robustness and informativeness of the signal contained in mitochondrial gene sequences for high-level phylogenetics have been questioned [33-35], despite the fact that they have been extensively used at virtually all taxonomic levels [36]. Specific criticism has focused on three major issues: a) the possibility to correctly model DNA or protein sequence changes; b) the impact of genome wide biases on phylogenetic reconstructions; c) outgroup choice.

While model-based methods of phylogenetic reconstruction for DNA sequences rely on quite sophisticated models of evolution [36], models of protein evolution have been more difficult to implement. Currently used matrices have been derived from nuclear (JTT [37]; WAG [38]), or vertebrate mitochondrial datasets (MtMam [39]; MtRev [40]), and are not therefore of immediate applicability to invertebrate mitochondrial data sets. This led to a reappraisal of the utility of these matrices [41,42] and the development of a more specific MtArt matrix based on arthropod mitochondrial genomes, that clearly outperforms other models across a variety of invertebrate data sets [41].

Genome-wide biases have been deemed responsible for some of the inconsistencies observed in mitochondrial genome trees. These include rate and base composition inequalities, that can lead to the attraction of long or AT-rich branches, and gene translocations on different strands, that impose a pressure towards the assimilation of within-strand base composition [43], thus violating model stationarity. These problems are generally addressed by excluding the most deviating sequences from the analysis, rather that attempting to correct for these violations. Therefore, the attention is shifted to the possibility of detecting, rather than correcting, these biases; though not addressing the problem directly, this is likely the best option available at present.

Finally, the opportunity of using different outgroups, and the dependency of the reconstruction upon outgroup choice, has been largely discussed for analyses encompassing all arthropods, whose closest relative is not known with confidence. On the other hand, in a study focusing on the pancrustaceans, outgroup choice naturally falls on myriapods and chelicerates.

In this study we analyzed all one-hundred currently available complete mitochondrial genomes from the Pancrustacea, including 5 newly sequenced genomes from the basal hexapod orders Collembola (4) and Microcoryphia (1), with the aim of resolving phylogenetic relationships among the Pancrustacea, and paying special attention to the position of basal hexapod lineages. Likelihood and Bayesian methods were used to analyze DNA and amino acid sequences. A new matrix of protein change was derived from the dataset itself, and its performance compared with other available matrices.

## Results

### Phylogenetic analysis of the nucleotide data set

**Figure 1:**
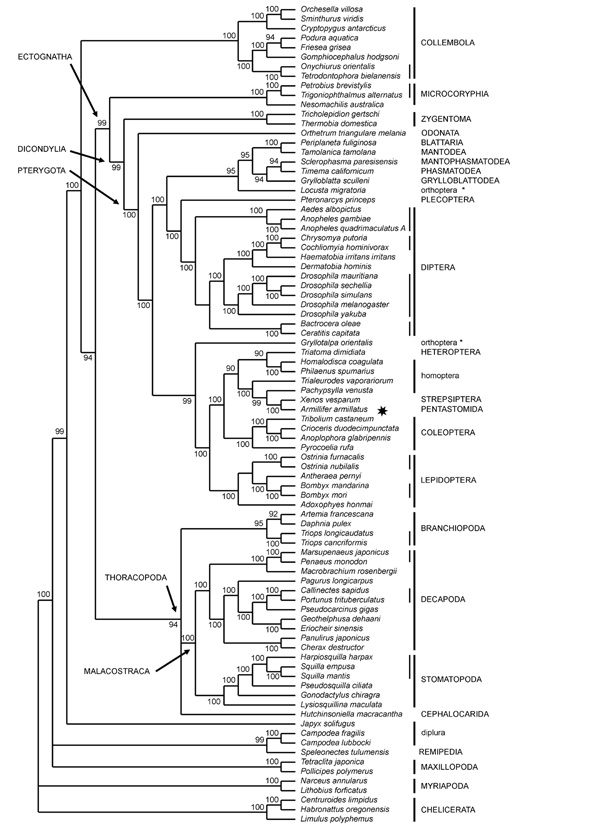
**Nucleotide tree, 1^st ^and 2^nd ^codon positions**. Numbers at nodes indicate posterior probabilities (×100). Vertical lines indicate monophyletic orders (thick) and families (thin). Lower-case order-level taxonomic names indicate non-monophyletic taxa.

In the Bayesian analysis of the nucleotide data set (1^st ^and 2^nd ^codon positions only) stationarity was found to be reached before 50,000 generations, and therefore 5% of sampled trees (500) were removed as the burnin of the analysis. Figure 1 shows the resulting tree, with posterior probabilities indicated at nodes.

In this tree, Hexapoda and Crustacea appear as mutually paraphyletic. Among crustaceans, major monophyletic lineages recovered are Cirripedia, Branchiopoda, Decapoda, Stomatopoda, and Malacostraca (Decapoda + Stomatopoda). Branchiopoda, Malacostraca and Cephalocarida form a monophyletic clade, that appears as the sister group of the Insecta. Cirripedia (Maxillopoda) are the basal lineage of the whole Pancrustacea grouping. Within the monophyletic Decapoda, supported formal taxa below the ordinal level are the Brachyura (*Callinectes*, *Portunus*, *Pseudocarcinus*, *Geothelphusa*, *Eriocheir*), the Dendrobranchiata Penaeidae (*Marsupenaeus *and *Penaeus*), and the family Portunidae (*Callinectes *and *Portunus*). The clustering of *Macrobrachium *with the two Dendrobranchiata makes the Pleocyemata paraphyletic (all Decapoda included here except for *Marsupenaeus *and *Penaeus*). Within Brachyura, the clustering of *Geothelphusa *with *Eriocheir *makes the Heterotremata paraphyletic. Phylogenetic relationships within the Stomatopoda reflect the accepted taxonomic scheme, with the three Squillidae (*Harpiosquilla*, *Squilla empusa *and *S. mantis*) clustered together.

The clustering of Branchiopoda, Malacostraca and Cephalocarida (Thoracopoda *sensu *[9,44]) with the Insecta, and with the exception of Collembola and Diplura, makes the Hexapoda, as traditionally defined, paraphyletic. Collembola are monophyletic, sister-group of the Thoracopoda + Insecta. Within Collembola, the superfamily Poduromorpha (*Podura*, *Friesea*,* Gomphiocephalus*, *Onychiurus*, and *Tetrodontophora*) and the family Onychiuridae (*Onychiurus *and *Tetrodontophora*) are monophyletic. Entomobryomorpha are paraphyletic with the symphypleonan *Sminthurus *nested within. Surprisingly, this analysis fails to recover the monophyly of Diplura. *Japyx solifugus *is basal to the cluster composed by Collembola, Thoracopoda and Insecta, while *Campodea fragilis *and *C. lubbocki *are associated with the remipedian *Speleonectes *in a basal branching of the Pancrustacea.

The monophyletic Insecta (apart from the position of *Armillifer*, see below) have monophyletic Microcoryphia, Zygentoma, Pterygota and Dicondylia (Zygentoma + Pterygota). Within Pterygota, whose basalmost lineage is the odonatan *Orthetrum*, the holometabolan orders Diptera, Lepidoptera and Coleoptera are monophyletic, but Holometabola *per se *are not. Traditional supraordinal assemblages, such as orthopteroids (Polyneoptera) and hemipteroids (Paraneoptera) are not recovered as monophyletic. A basal split distinguishes two major clusters of Neoptera. The first one is composed by the representatives of polyneopteran orders, the Plecoptera (*Pteronarcys*), and the Diptera. Within Polyneoptera, with the basal *Locusta *(Orthoptera), the clade (Blattaria + Mantodea) is sister group to the assemblage (Grylloblattodea + (Mantophasmatodea + Phasmatodea)). Highly unusual features are the disjunction of the orthopteran genera *Locusta *and *Gryllotalpa*, and the association of the pentastomid *Armillifer *with the strepsipteran *Xenos*, these latter nested within the clade of hemipteroid species (Heteroptera + Homoptera). This clade is sister to the Coleoptera and both are joined with the Lepidoptera in the second major clade of Neoptera.

### A new model of amino acid replacement in Pancrustacea: MtPan

A model describing the evolution in time of the sequences can be built empirically using properties calculated through comparisons of observed sequences, or parametrically using chemical and biological properties of DNA and amino acids. When properties shared by a set of sequences are too subtle or hidden to be analytically represented (or there are too many degrees of freedom), amino acid replacement models should be obtained through an empirical approach. This approach has the advantage of allowing a small number of degrees of freedom since parameter values are fixed, being estimated only once and then assumed to be applicable to all datasets. The result is a model computationally easy to use, but the breadth of the applicability has to be considered carefully because there is little or no way for it to be influenced by the data analysed.

### Phylogenetic analysis of the amino acid data set

**Figure 2:**
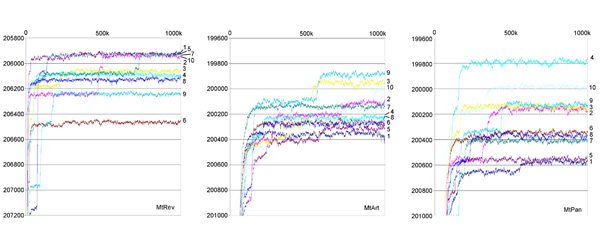
**Plots of likelihood vs generations**. Generations (1 to 1,000,000) are reported on the x-axis. -Log likelihood values are reported on the y-axis. Numbers refer to each of the 10 runs performed with each matrix.

The amino acid data set (86 taxa, 3006 amino acid aligned positions) was analyzed with three different matrices of amino acid replacement using MrBayes. The 10 analyses run for each matrix were compared in plots where the Log likelihood of the sampled trees are plotted against generations (Figure 2).

In the analyses with the MtRev matrix, the Log likelihood values of the final topology of the 10 runs ranged from -206,500 to -205,900, with five independent runs (#1, #2, #5, #7, #10) converging to a very similar score (around -205,900). In 3 of these 5 runs (#1, #2, #10), stationarity was reached relatively early (before the 150,000^th ^generation), while runs #2 and #7 suddenly jumped to a better likelihood score after 500,000 and 750,000 generations, respectively. In this case, a consensus tree was constructed pooling together all sampled trees from the five runs, after the removal of burnin as follows: 1,000 trees for run #5 (10%), 1500 trees for runs #1 and #10 (15%), 5,000 trees for run #2 (50%) and 7,500 trees for run #7 (75%).

In the analyses with the MtArt matrix, the Log likelihood values of the final topology of the 10 runs ranged from -200,400 to -199,900, and two runs, #3 and #9, converged to a similar score. Interestingly, these two runs reach stationarity late in the generations, jumping to the higher score after 550,000 generations (55% of burnin). We therefore constructed the consensus tree using all trees sampled in both runs, after removal of the burnin (5,500 trees).

In the 10 runs with the MtPan matrix, the Log likelihood of the final topology ranged from -200,600 to -199,800, with one run (#4) converging to a considerably better likelihood score than all other runs. In run #4, stationarity was reached after about 200,000 generations, so the burnin removed to build the final consensus tree was 2,000 trees (20%).

The three reconstructions and likelihood plots obtained using different matrices were compared. The matrix MtPan apparently outperforms the two other matrices with this data set, as expected, as it gives higher likelihoods for most runs, and the resulting trees are more resolved and display higher posterior probabilities at most nodes. Furthermore, being the matrix MtPan specifically developed based on Pancrustacean sequences, it is likely to model evolutionary processes with more accuracy in this specific data set than other matrices developed for different purposes. The following considerations are based on the run (#4) from matrix MtPan that converges to the higher likelihood.

**Figure 3:**
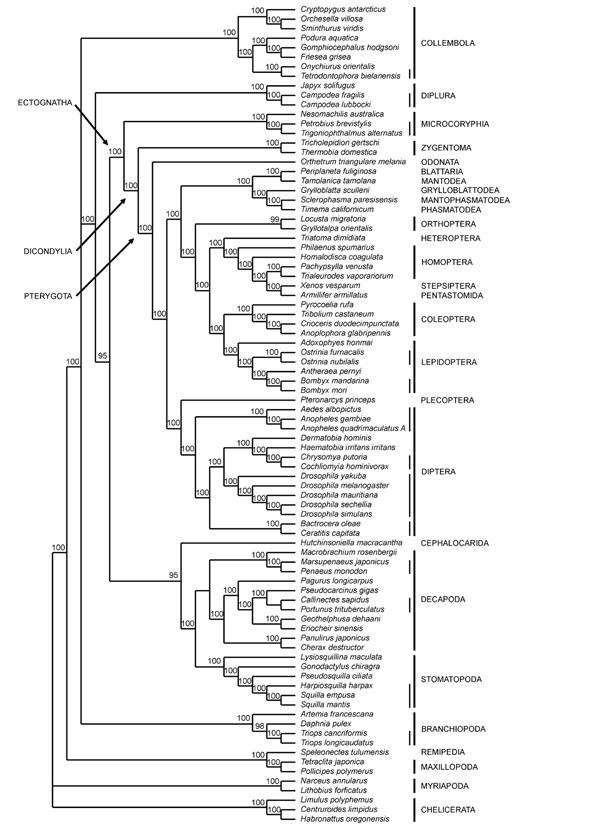
**Amino acid tree, MtPan model**. Number at nodes indicate posterior probabilities (×100). Vertical lines indicate monophyletic orders (thick) and families (thin).

The phylogenetic tree obtained (Figure 3) retrieves the following recognized taxa as monophyletic: Collembola, Diplura, Insecta, Microcoryphia, Zygentoma, Dicondylia, Pterygota, Diptera, Lepidoptera, Coleoptera, Malacostraca, Decapoda, Stomatopoda, Branchiopoda and Cirripedia. Conversely, both Hexapoda and Crustacea are retrieved as paraphyletic. The Malacostraca + *Hutchinsoniella *clade is the sister-group of all Insecta, with the exclusion of Diplura and Collembola. Within Insecta, the three more intensively sampled orders of Holometabola (Lepidoptera, Coleoptera, and Diptera) are monophyletic, so are the basal taxa Microcoryphia and Zygentoma, but the polyneopterans and paraneopterans do not form monophyletic clusters, nor do the Holometabola. The plecopteran *Pteronarcys *clusters with the Diptera in a basal clade, and the representatives of the remaining orthopteroid orders (*Periplaneta*, *Tamolanica*, *Sclerophasma*, *Timema*, *Grylloblatta*, *Locusta *and *Gryllotalpa*) do not cluster together. The two orthopterans (*Locusta *and *Gryllotalpa*), in fact, cluster with a clade joining Coleoptera + Lepidoptera, and a group of hemipteroid species (*Triatoma*, *Pachypsilla*, *Trialeuroides*, *Homalodisca *and *Philaneus*), with the strepsipteran *Xenos *and the pentastomid *Armillifer *nested within. In this group, *Triatoma*, the only Heteroptera, is basal to all other homopterans. Within Collembola, the relationships are the same as those derived form the nucleotide tree, with the exception of the closer affinity of *Friesea *with *Gomphiocephalus *rather than with *Podura*. Among crustaceans, relationships within Malacostraca (here, Decapoda + Stomatopoda) are the same as those observed in the nucleotide tree, and largely congruent with the known phylogeny of the group. The two Cirripedia (Maxillopoda) cluster together, as do the four Branchiopoda. However, Branchiopoda do not cluster with Malacostraca, as they do in the nucleotide tree, but come out of a well supported basal tricotomy with Collembola and (Insecta + (Malacostraca + *Hutchinsoniella*)).

## Discussion

In the analysis of the amino acid data set, the plots of likelihood values versus generations (Figure 2) allow the evaluation of the robustness of the results with respect to differences among runs and variations of starting points. In this context, while in the analysis using the MtPan matrix, one run (#4) selects a clearly better topology than all other runs, in the analyses using the MtArt and the MtRev matrices, several runs converge to similar optimal topologies (2 for MtArt and 5 for MtRev). A detailed scrutiny of the topologies selected with each matrix (not shown), and the lower resolution of the reconstructions resulting from the consensus of the trees sampled in each run, pooled for each matrix, show that topologies with similar likelihood values may differ considerably. That is to say that considerably different topologies may have similar likelihood values. In addition, the differences are generally concentrated in the deepest nodes, rather than the most apical ones. This suggests that relationships among basal lineages of Pancrustacea, based on the amino acid sequences of mt PCGs, are still quite unstable, and that the choice of one topology over another may be dependent on the efficiency of the algorithm to explore the likelihood space, as well as on the alignment, data and taxon choice, and the matrix of amino acid replacement. Nevertheless, the use of a taxon-specific model of evolution (MtPan) may significantly improve the performance of the analysis, and the tree obtained with the best run using MtPan is considerably better (harmonic mean of -Log likelihood = 199848.98) than all other best trees obtained with the two other matrices (harmonic mean of -Log likelihood always >200,000.00). We therefore consider the topology of Figure 3 as our best estimate of the phylogenetic relationships of the Pancrustacea using mitochondrial PCGs, and focus the following discussion on this topology.

The trees obtained based on the nucleotide dataset (complete and with 3^rd ^codon positions excluded) are largely congruent among them and with the aforementioned MtPan tree #4. Comparing the two nucleotide trees, the one obtained based on the complete dataset displays generally lower posterior probabilities, especially at the deeper nodes, than the one obtained based on 1^st ^and 2^nd ^codon positions only. This is likely due to the fact that 3^rd ^codon positions, in comparisons among more distant sequences, are highly saturated, and therefore tend to blur the phylogenetic signal at deeper nodes, hence the decrease in posterior probabilities. We regard the analysis on 1^st ^and 2^nd ^codon positions as our best estimate for the nucleotide dataset, and refer to this in all subsequent reasoning.

The most remarkable outcome of the analyses presented here is the supposed reciprocal paraphyly of Crustacea and Hexapoda, already suggested in previous studies [15,16,32], and here confirmed on the basis of the largest data set available so far for mitochondrial genomes of Pancrustacea.

In both trees the Cirripedia (Maxillopoda) occupy a basal position, and in the analysis with the amino acid sequences they are clustered with the remipedian *Speleonectes*. Remipedians are a recently discovered arthropod taxon, whose phylogenetic position is still debated [31,45-47]. They have been considered the most basal group of crustaceans [48], but according to their complex brain architecture, they are somehow associated with Malacostraca and Insecta [49]. Other genes also suggest a closer affinity of Remipedia with Cephalocarida (*Hutchinsoniella*) and the Insecta [7,18,46]. The placement of Remipedia and Maxillopoda at the base of the pancrustacean tree conflicts with gene order data that include Cephalocarida, Maxillopoda and Pentastomida in a more derived position [16,31]. Other phylogenetic studies based on nuclear genes, or on a combined analysis of molecular and morphological data, support a closer relationship between Maxillopoda + Malacostraca [18,50].

In the analysis of the nucleotide data set, *Speleonectes *is clustered with the two species of the dipluran genus *Campodea*. Although Diplura have been previously suggested as being paraphyletic [27], this was based on the comparison of the structure of the ovary with respect to other entognathan hexapods. In this context, the unusual association of *Campodea *and *Speleonectes *is most likely due to anomalies in the evolution of the molecules, such as uneven rates of substitution and/or attraction of long branches. Diplurans have already been found to display accelerated rates of evolution leading to long branches that may uncoventionally attract unrelated taxa [30,47,50,51]. Remarkably, Diplura form a well supported monophyletic clade based on the analysis of the amino acid data set.

Regardless of the mono/paraphyly of Diplura, their position, as well as the position of Collembola, in both trees, strongly suggests non-monophyly of Hexapoda, as commonly defined. Although traditionally included in the Entognatha [21], recent studies and the re-interpretation of morphological characters have challenged the common origin of entognathy observed in living Collembola, Protura and Diplura [22,24,25,52,53]. Moreover, molecular phylogenetic studies have provided alternative views of internal relationships of Entognatha. Nuclear genes (mostly rDNA genes) usually join Diplura and Protura in the Nonoculata, and place Collembola as the basal taxon of Entognatha [17,26,29,30,50], although always in the context of monophyletic Hexapoda. On the other hand, previous studies based on the mitochondrial genome consistently place Collembola outside the clade joining the Insecta with some crustacean lineages (Malacostraca and, sometimes, also the Branchiopoda) [15,16,19,32]. Our study confirms this view, and the addition of Diplura reinforces the idea of a paraphyletic Hexapoda. Non-monophyly of Hexapoda is indeed difficult to accept from a morphological perspective [28], and the robustness of the reconstructions based on mitochondrial data sets available so far has been challenged as a potential artifact of the analysis: insufficient sampling density, gene selection, outgroup choice, alignment, type of data, analytical methods, and peculiarities of the structural evolution of the mitochondrial genome [33-35]. In addition, most molecular data sets based on nuclear genes also support hexapod monophyly [17,23,29], but not all of them, especially when mitochondrial and nuclear genes are combined [26]. In our study, we have tried to tackle some of the most common criticism by extending our data set to all mitochondrial protein coding genes, and adding more dipluran and collembolan sequences, as well as exploiting the higher number of pancrustacean sequences now available. To improve the phylogenetic analysis we also used two new matrices of amino acid replacement [21], including one specifically designed for Pancrustacea (MtPan).

There is a growing body of evidence that phylogenetic inferences are more reliable the more accurate the model of sequence evolution are and that maximum likelihood or posterior probability represent a robust criterion for the choice of the best models. MtArt and MtPan models are both derived from the analysis of inferred substitutions in reference sequences, therefore they have fixed and equal number of parameters. Advantages of this approach can be the better description of the evolution of the sequences under study, if a suitable reference set is used, particularly if this reference set is large. Disadvantages can be inaccuracy owing to an inappropriate reference set and a lack of a broader biological interpretability of purely empirical findings. Here we use the sequence data set under study to derive a model to best to accomodate the trade-off between incorporating into models enough biological reality to capture evolutionary information accurately and avoid overparameterization that can lead to a loss of discriminatory power. The differences between MtPan and MtArt, although small, are subtly widespread in all 20 × 20 amino acid exchange rates and frequencies and the Mantel test, which computes a correlation between two n × n distance or similarity matrices, shows that the two matrices are significantly different.

The consistent finding of non-monophyletic Hexapoda requires some considerations. The most evident outcome of this result is that the character "hexapody", traditionally invoked as the most important synapomorphy of Hexapoda, may have arisen at least twice during arthropod evolution. This may even have happened in marine environments [54], rather than as an adaptation to terrestrialization. This implies that Collembola, Diplura and Insecta could be better regarded as independent lineages evolved from different crustacean ancestors after terrestrialization [18].

While Collembola and Diplura appear to stem out the pancrustacean clade very early, and no clear relationship has yet been established with any crustacean lineage, our mitochondrial data set suggests that the Insecta could be more closely related, among the Crustacea, with the Malacostraca and, possibly, the Branchiopoda. In this context, our analyses of the nucleotide and amino acid data sets differ. In the nucleotide data set, the Branchiopoda are part of a well supported unresolved trichotomy with Malacostraca and the cephalocaridan *Hutchinsoniella*. This clade is the sister taxon of the Insecta, and corresponds to the Thoracopoda. Relationships within Thoracopoda remain debated and focus on the homologous patterns of the limb structure (hence the name thoracopods = appendages of the thorax). In this respect, the presence of a single epipod per thoracopod have been proposed as a potential synapomorphy shared by Malacostraca and Branchiopoda (and possibly Cephalocarida) [9], although alternative structures and functions of epipods, found in different crustacean groups, may have led to erroneous identification of homologous patterns. In the amino acid data set, the sister taxon of the Insecta is limited to Malacostraca + Cephalocarida, with the Branchipoda emerging earlier in the tree. Regardless of their closest crustacean relative(s), all analyses here performed support the monophyly of the Insecta *s.s*. (Ectognatha), although relationships among their internal lineages deserve a careful scrutiny.

Reflecting the most widely accepted interpretation, based on morphological and molecular data [17,22,29,55], the basal splitting of the Insecta separates the Microcoryphia (bristletails) from the Dicondylia (Zygentoma + Pterygota), although some recent analyses of other nuclear markers [18] would suggest resurrecting the long-abandoned Thysanura *s.l*. (Microcoryphia + Zygentoma). Within Microcoryphia and Zygentoma (silverfish), relationships are stable across different analyses, and congruent with the accepted taxonomy. The odonatan *Orthetrum *is the basal lineage of the Pterygota (or Metapterygota *sensu *[56]). The clustering Odonata + Neoptera is also supported by several morphological features [55,57], and, in particular, by the complete fixation of the anterior articulation of mandibles [58], but the absence of the Ephemeroptera from our analysis prevents from drawing conclusions.

This mitochondrial data set dramatically differs from most widely accepted reconstructions when looking at the phylogeny of Neoptera. On one hand, the two analyses provide strikingly different results. On the other hand, no major traditional lineage, except for the monophyly of Lepidoptera, Coleoptera and Diptera, seems to be recovered. The most evident anomaly is the placement of the pentastomid *Armillifer*, a putative crustacean potentially associated with Maxillopoda and Cephalocarida [31], which is joined with the strepsipteran *Xenos *in a derived position among Pterygota. This quite evident artifact of the analysis may be due to exceptionally high rates of evolution shared by these two sequences, a phenomenon possibly affecting also the clustering of *Xenos *and *Armillifer *with the five hemipteran sequences. The representatives of 7 polyneopteran orders are included in our analysis. However, the plecopteran *Pteronarcys *always clusters with the Diptera, somehow confirming an earlier claim by Hennig [21] that no conclusive evidence is available of the inclusion of Plecoptera in the Polyneoptera (Paurometabola *sensu *[59] plus Plecoptera). Another relevant case is that of Orthoptera, here represented by *Locusta *and *Gryllotalpa*, which either do not cluster together (Figure 1), or do not cluster with the remaining polyneopterans (Figure 3). The internal relationships among these latter orders (Blattodea, Mantodea, Mantophasmatodea, Phasmatodea and Grylloblattodea) match the results obtained using a similar data set [60], with a monophyletic Dictyoptera (Blattaria + Mantodea), and a relationship between Mantophasmatodea and Phasmatodea, with the Grylloblattodea as their sister group, therefore rejecting the Xenomomia [61]. The taxonomic status and phylogenetic position of the recently discovered order Mantophasmatodea in the context of the polyneopteran assemblage has been thoroughly discussed on morphological and molecular grounds [60-63], and is beyond the scope of our present analysis. The relationships of polyneopteran insects remains unsolved and may have been obscured by severe extinction events [64] or by the lack of good synapomorphic characters.

Surprisingly, the Holometabola do not form a monophyletic clade, in open disagreement with most morphological [21,65] and molecular [66] analyses. The failure of this data set to support the Holometabola may be due to the biased sampling of this taxon, with many major lineages (orders) still missing from the analysis (i.e.: Mecoptera, Siphonaptera, Trichoptera, Neuroptera, as well as the Hymenoptera, not included in the analysis due to their extreme nucleotide compositional bias), while some (i.e.: Diptera, Coleoptera, Lepidoptera) being represented by many species. Within the monophyletic Coleoptera, phylogenetic relationships are congruent with the traditional taxonomy: the two Chrysomeloidea (*Crioceris *and *Anoplophora*) cluster together, and, with the Tenebrioidea (*Tribolium*), representing the Cucujiformia, to the exclusion of the Elateroidea *Pyrocoelia*. Also within Lepidoptera, relationships reflect the accepted taxonomy, with the basal Tortricoidea (*Adoxophyes*), and the clade of Obtectomera clustering Bombycoidea (*Bombyx *plus *Antherea*) and Pyraloidea (*Ostrinia*). Finally, both trees support the basal dipteran split between the nematocerans Culicidae (*Aedes *and *Anopheles*), and the brachycerans. Within Brachycera, our analysis fails to recover the taxon Acalyptrate (which should include, here, Drosophilidae and Tephritidae) by joining the Drosophilidae with the remaining Calyptrate.

One interesting feature of our study is the inclusion in the analysis of 8 species from 7 different families of Collembola, allowing a preliminary phylogenetic reconstruction of inter-familiar relationships. In both our trees, the Collembola are monophyletic, and they differ only for the relative position of the three poduromorph species *Friesea*, *Gomphiocephalus *and *Podura*. Traditional recognized groupings of Collembola are Neelipleona, Symphypleona and Arthropleona, the latter furtherly divided into Entomobryomorpha and Poduromorpha [67-69]. One remarkable outcome of our analysis, is the nesting of the Symphypleona within the Entomobryomorpha, making the Arthropleona paraphyletic, and confirming the conclusions of D'Haese [69]. Our analysis also supports the monophyly of the Poduromorpha (Poduridae, Hypogastruridae, Neanuridae, Onychiuridae, only to mention the families represented in our data set), defined by the presence of a well developed protergite, with the critical genus *Podura *being part of this taxon. This contrasts with the interpretation that the hypognathous position of the head is the synapomorphy uniting Poduridae (+ Actaletidae) with the Symphypleona [70]. Although rejected on the basis of heart morphology [71], the association between Poduridae and Symphypleona (+ Neelipleona) was resurrected by Moen and Ellis [68]. Finally, the nesting of *Podura *within the monophyletic Poduromorpha was strongly supported also by the most recent morphological and molecular (with nuclear genes) analyses [69,72]. As expected, the two onychiurids, *Onychiurus *and *Tetrodontophora*, cluster together. Their strict relationship is supported also by gene order data, given that both species share the unique mitochondrial translocation of the *trnS*^*uga *^from the original position between *cob *and *nad1 *to a new location between *trnI *and *trnM *[16,73].

Concerning shallow relationships in crustacean clades, the two trees are perfectly congruent regarding the phylogeny within Stomatopoda and Decapoda, but not within Branchiopoda. However, the relationships among Stomatopoda significantly differ from those proposed on morphological grounds [74], which group the Lysiosquillidae (here represented by *Lysiosquilla*) with the Squillidae (here: *Squilla *and *Harpiosquilla*), and the Gonodactylidae (here: *Gonodactylus*) with the Pseudosquillidae (here: *Pseudosquilla*). Nevertheless, the biodiversity of Stomatopoda is so underrepresented in our study that molecular phylogenetic relationships might still be unstable. Within the Decapoda, with 10 species from 8 different families sampled, the basal Dendrobranchiata (*Marsupenaeus *and *Penaeus*) cluster with *Macrobrachium*, traditionally considered a basal lineage of the Pleocyemata [75]. In the remaining Pleocyemata, the clustering of Palinura (*Panulirus*) with Astacidea is in agreement with morphological data [75], but *Pagurus *(Anomura) is clustered with the monophyletic Brachyura (*Callinectes*, *Portunus*, *Pseudocarcinus*, *Geothelphusa*, *Eriocheir*) instead of the clade Palinura + Astacidea. Within Brachyura, a partial conflict exists between molecular data among the Heterotremata: in fact, *Callinectes*, *Portunus*, *Pseudocarcinus *and *Geothelphusa *share the translocation of the *trnH *in a new position between *trnE *and *trnF*, but the reconstruction of the phylogenetic relationships based on sequence analysis places *Geothelphusa *with *Eriocheir*, which does not share the translocation of *trnH *in the same position. Finally, within Branchiopoda, the basal position of the anostracan *Artemia*, expected on morphological grounds [76], is retrieved only in the amino acid tree.

## Conclusion

Using the largest available mitochondrial DNA data set for Pancrustacea, our present study confirms that phylogenetic analyses based on the sequence of the mitochondrial protein coding genes consistently support the reciprocal paraphyly of Hexapoda and Crustacea. While the Insecta *s.s*. are shown as a robust monophyletic clade, Collembola and Diplura fail to be clustered with the remaining Hexapoda. On the other hand, some lineages of crustaceans, namely Malacostraca, Cephalocarida and, possibly, Branchiopoda are the sister taxon of the Insecta. This reconstruction supports an evolutionary scenario in which hexapody may be considered as the results of independent events of terrestrialization occurred in different lineages of crustacean-like ancestors. If this hypothesis was true, then finding the closest crustacean relative to each hexapod lineage becomes the next major challenge, which implies sampling the diversity of crustaceans in a much more massive way. It also implies that the third lineage of entognathan hexapods, the Protura, needs to be included in future analyses, in order to represent also the complete diversity of basal hexapods. In addition, we show that the use of a taxon-specific matrix of amino acid replacement helps improving the performance of the phylogenetic reconstruction using amino acid sequences of mitochondrial PCGs.

## Methods

### Data set and alignment

All available complete mitochondrial genomes from Pancrustacea were used in this study. The monophyly of Pancrustacea was taken as granted, being this taxon consistently supported in a variety of molecular studies based on mitochondrial and nuclear DNA [6,10,18].

The AMIGA database [77] was used to assemble the initial dataset. All complete mitochondrial genomes of pancrustacean species available in GenBank in July 2006 (RefSeq only) were retrieved, totalling 95 sequences. In addition, five sequences from Myriapoda and Chelicerata were added as outgroups. The nucleotide sequences of individual protein coding genes (PCGs) were downloaded and clustered in 13 separate files. Five new undescribed complete mitochondrial genomes determined in our laboratory (4 Collembola and 1 Microcoryphia) were also added. This generated a complete data set of 105 sequences (100 Pancrustacea and 5 outgroups, listed in Additional File 1).

The nucleotide sequences of each PCG were retro-aligned using the RevTrans 1.4 server available through the DTU-CBS website [78]. Both the amino acid and the corresponding nucleotide alignments were retained. Finally the 13 data set were concatenated, species-by-species, to produce a final alignment of 105 sequences, 12552 nucleotide and 4184 amino acid positions (alignments available upon request).

The amino acid data set was manually inspected to isolate areas of unreliable alignment. These, and the corresponding positions in the nucleotide dataset, were flagged and excluded from the analysis. A total of 28% of aligned positions were removed, with some genes more affected by the elimination (*atp8 *and *nad6*, over 60%), and others less affected (*cox1*, *cox3*, *atp6 *and *cytb*, less than 10%).

Each sequence was subsequently examined in order to identify those characteristics that have been reported to introduce errors in the phylogenetic reconstruction. These include extreme compositional bias [34,35], inversion or translocation of genes on opposite strand [35], inversion of control region [43], lack of *atp8*. This procedure led to the exclusion of 19 sequences (17 hexapods and 2 crustaceans), reducing the data set to 86 taxa. All following analyses were performed on this final data set, consisting of 86 taxa, 9018 nucleotide and 3006 amino acid aligned positions.

### Matrix of amino acid replacement

Here we have followed an approach proposed by David Jones, Willie Taylor and Janet Thornton [37]. Our model is a Markov process model, defined by a 20 × 20 matrix containing the relative rates (i.e. the relative numbers, on average and per unit time) of occurrence of all possible replacements derived simply by counting observed amino acid replacements in the pancrustacean sequence databases. Only very closely related sequences (85%) were considered, to reduce the frequency with which observed replacements (e.g. A⇒S), were in fact the result of a set of successive unobserved replacements (e.g. A⇒R⇒S). From this matrix are calculated the probabilities of change from any nucleotide to any other nucleotide (or any amino acid to any other amino acid), including the probability of remaining the same, over any period of evolutionary time (e.g. from one end of a branch to the other) at any site.

### Phylogenetic analysis

Phylogenetic analysis was performed on both nucleotide and amino acid sequences using a bayesian approach as implemented in MrBayes, ver. 3.1 [79,80]. For nucleotide sequences, the GTR+I+G model of sequence evolution was used. One million generations were run, with four MC chains, and trees were sampled every 100 generations. Two independent analyses were run for the complete data set (9018 nucleotides) and on 1^st ^and 2^nd ^codon positions only (6012 nucleotide positions). The Log likelihood scores of each sampled tree were plotted against generations in order to assess the number of generations needed to reach stationarity and to evaluate the appropriate burnin (50,000 generations; see Results). A consensus topology of all trees, after the removal of burnin, was constructed using PAUP* ver. 4.0b10 [81], with the percentage of trees where each node was found expressed on the tree as posterior probabilities.

For amino acid sequences, three different matrices of amino acid replacement were used, and the results compared: 1) the general matrix available for mitochondrial genomes, but based on vertebrate taxa, MtRev [40], MtArt [41], and our specifically developed matrix, MtPan. Ten independent runs, of one million generations, two MC chains, with different random starting points were performed for each matrix. Trees were sampled every 100 generations. Log likelihood scores of the trees in all runs for each matrix were plotted against generations. Three plots, one for each matrix, were thus obtained, allowing to assess the rate of variability of the different runs for each matrix and the appropriate burnin. Final topologies for each matrix were obtained from the best run (the one converging to the best likelihood score), or from a group of runs, if more than one run were found to converge to nearly identical likelihood scores.

## Competing interests

The authors declare that they have no competing interests.

## Authors' contributions

AC sequenced the five new mitochondrial genomes and drafted the manuscript. PL assembled the new matrix of amino acid replacement and directed the phylogenetic analysis of the amino acid data set. FN assembled the data set, aligned the sequences and assisted with the phylogenetic analysis. EvdW carried out the analyses with the amino acid data set. FF directed the research, performed the phylogenetic analysis with the nucleotide data set, and produced the final manuscript. All authors read and approved the final manuscript.

## Supplementary Material

Additional file 1 **List of taxa used in this study**. Taxa in boldface are included in the analysis. Taxa not in boldface have been removed on the basis of the following justifications: ^1 ^– extreme AT bias according to [34,35]; ^2 ^– inversion or translocation on the opposite strand of PCGs [35]; ^3 ^– inversion of CR [43]; ^4 ^– lacks ATP8 gene; ^5 ^– AT content >80%. Click here for file
